# Hierarchical self-assembly of aromatic peptide conjugates into supramolecular polymers: it takes two to tango

**DOI:** 10.1039/d1sc05589e

**Published:** 2021-12-10

**Authors:** Maëva Coste, Esteban Suárez-Picado, Sébastien Ulrich

**Affiliations:** IBMM, Institut des Biomolécules Max Mousseron, CNRS, Université de Montpellier, ENSCM Montpellier France sebastien.ulrich@enscm.fr

## Abstract

Supramolecular polymers are self-assembled materials displaying adaptive and responsive “life-like” behaviour which are often made of aromatic compounds capable of engaging in π–π interactions to form larger assemblies. Major advances have been made recently in controlling their mode of self-assembly, from thermodynamically-controlled isodesmic to kinetically-controlled living polymerization. Dynamic covalent chemistry has been recently implemented to generate dynamic covalent polymers which can be seen as dynamic analogues of biomacromolecules. On the other hand, peptides are readily-available and structurally-rich building blocks that can lead to secondary structures or specific functions. In this context, the past decade has seen intense research activity in studying the behaviour of aromatic-peptide conjugates through supramolecular and/or dynamic covalent chemistries. Herein, we review those impressive key achievements showcasing how aromatic- and peptide-based self-assemblies can be combined using dynamic covalent and/or supramolecular chemistry, and what it brings in terms of the structure, self-assembly pathways, and function of supramolecular and dynamic covalent polymers.

## Abstract 2

The dynamic combination of aromatics and peptides yields functional supramolecular biopolymers which self-assemble hierarchically and adapt through non-covalent interactions and/or reversible covalent reactions.

## Graphical abstract

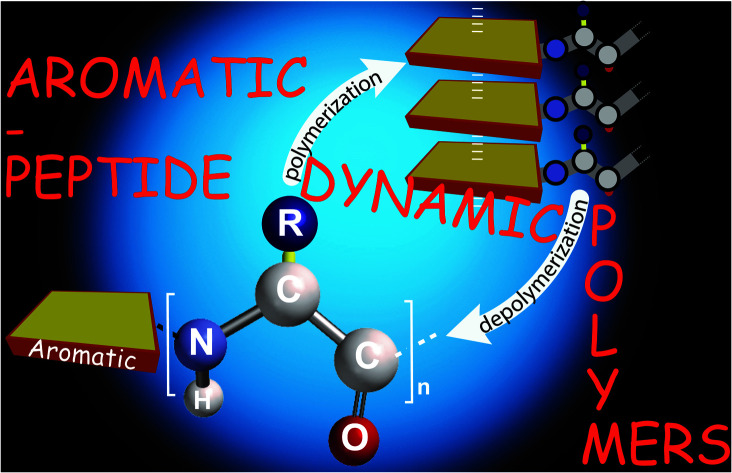

## 1. Introduction

Amino acids are the monomers that make up proteins, which perform vital functions in living organisms. The recognition and catalytic functions of proteins greatly depend upon their composition and sequence, folding, and multivalent interactions – all of which are encoded by the precise insertion of amino acids. Mainly, peptides play this role by triggering supramolecular hierarchical self-assembly, from β-sheets and α-helixes up to quaternary assemblies, with potential stimuli-responsive behaviour due to the reversibility of non-covalent interactions. Exploiting this principle, peptides have been recently introduced into building blocks that yield, by bottom-up self-assembly, macromolecules such as supramolecular polymers or dynamic covalent polymers. Due to their expressed multivalency,^1,2^ dynamic covalent polymers, as well as supramolecular polymers have become useful tools for the biomolecular recognition of proteins^3,4^ and nucleic acids,^5–7^ for (targeted) delivery,^8–11^ bioimaging,^12^ and applications in catalysis.^13,14^ This strategy of inserting peptides into self-assembled polymers enables generation of dynamic analogues of proteins which, thanks to the reversibility of non-covalent interactions or covalent reactions, can grow and collapse depending on the conditions and exchange building blocks/sequence, all of which greatly contribute to adapting their functions. Such an approach bears a strong degree of biomimicry for accessing supramolecular biomaterials that resemble some of the biopolymers found in the cytoskeleton such as actin microfilaments,^15^ and are currently considered to have strong potential toward functional smart polymers.^16^ Thus, the intertwining of the area of supramolecular polymer chemistry with the realm of peptide chemistry can be a fruitful route toward biomimetic smart materials. Indeed, the synergistic combination of multiple supramolecular interactions afforded by different types of building blocks (*e.g.* hydrogen bonding, π–π interactions, *etc.*) has been recognized as a powerful strategy for achieving a bottom-up hierarchical self-assembly.^17^ In this review, we will summarize the achievements that have been reported in the last decade when inserting peptides into both supramolecular and dynamic covalent polymers, either for endowing them with specific structures and/or functions, or for impacting their complex self-assembly pathways.

## 2. Effects of aromatics and peptides on dynamic covalent polymers

### 2.1. What are dynamic covalent polymers?

**Scheme 1:**
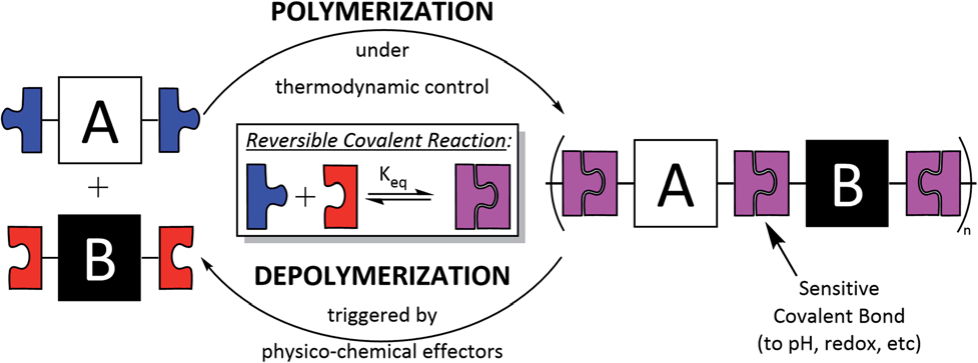
Principle of formation of dynamic covalent polymers (DCPs) using reversible covalent reactions. The example depicted here features linear alternating DCPs made of complementary bifunctional building blocks A and B.

Dynamic covalent polymers (DCPs) are macromolecules obtained through dynamic covalent self-assembly processes that rely on the use of reversible covalent reactions.^18–21^ Unlike classical polymers, DCPs are therefore dynamic objects which can reversibly form and fall apart, and adapt their constitution (length and sequence) to internal (*e.g.* folding) and/or external (pH, redox, and presence of a binding partner) forces (Scheme 1). DCPs can be multidimensional, from linear 1D to cross-linked 3D and different types of DCPs have been reported to date: poly-imines, poly-acylhydrazone,^22^ poly-oximes,^23,24^ and poly-disulphides^25^ to cite a few.

Since they are formed under thermodynamic control in an equilibrium polymerization, DCPs are polydisperse (PDI ≈ 1.5–2), and they often reach a plateau in terms of the size and degree of polymerization (typically an upper limit of DP ≈ 10–20), which probably represents the most important limitation of DCPs. In an isodesmic equilibrium polymerization (equal equilibrium constant *K*_eq_ for each step), *K*_eq_ limits the conversion *ρ* (*ρ* = *K*_eq_^1/2^/(1 + *K*_eq_^1/2^)) which dictates the degree of polymerization according to Carothers' equation (DP = 1 + *K*_eq_^1/2^).^26^ Typically, this means that, in a closed system, a DP of 100 can only be achieved with K equal to 10^4^. Moreover, as in classical step-growth polymerization, the stoichiometry balance should be near ideal in order to reach a high degree of polymerization. However, when cooperative effects come into action, meaning that equilibrium constants increase throughout the multi-step polymerization process, then very long DCPs can be formed, even under conditions of imbalanced stoichiometry.^27^ Such cooperative effects can originate from internal (*e.g.* activation/deactivation^28,29^ and folding^30,31^) or external (*e.g.* template-assisted polymerization^32^) forces that modulate reactivity throughout the polymerization process. These secondary interactions can greatly assist the polymerization process, such as the π–π stacking interactions that are commonly seen in supramolecular polymerization involving aromatic compounds.

DCPs have already found numerous applications in materials science due to their self-healing and shape memory properties^19,33^ and are the current subject of growing interest in biological sciences, for instance, as smart and responsive delivery vectors.^8–10^

### 2.2. Functional DCPs by peptide insertion

**Fig. 1:**
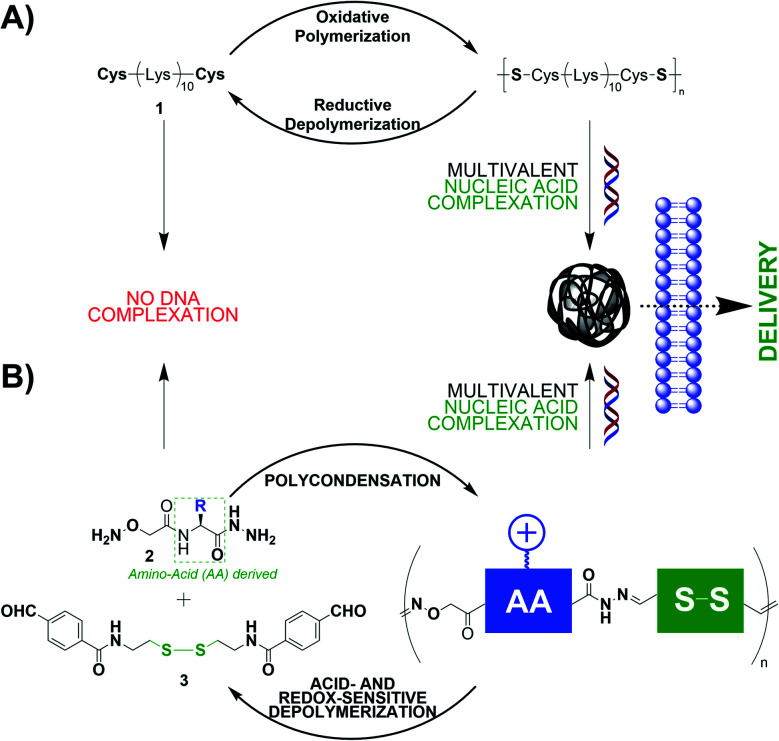
Dynamic covalent polymers endowed with the function of nucleic acid recognition which is encoded by inserted cationic amino acids.

Disulphides are redox-sensitive covalent bonds commonly found in proteins. The access to poly-disulphide DCPs can be achieved using an amino acid: cysteine. Indeed, peptides appended at both C and N termini with cysteine would lead to linear DCPs through such an oxidative polymerization.^34^ The group of Seymour reported early that the cationic peptide 1 – a Lys_10_ peptide flanked with one cysteine at each end, leads, through a DMSO-promoted oxidative polymerization, to DCPs able to complex and deliver plasmid DNA (pDNA) thanks to multivalent electrostatic interactions (Fig. 1A).^35^ Thee DCPs were formed by step-growth polymerization and have molecular weights reaching 187 000 Da. In this example, one amino acid (Cys) is responsible for generating the DCPs while the others (Lys) carry out the function of complexing nucleic acids through electrostatic interactions. More recent designs used cyclic disulphides that undergo DNA-templated polymerization through ring-opening polymerization.^36^

Our group studied another class of DCPs for gene delivery applications: poly-acylhydrazones. After having established the ability of cationic poly-acylhydrazones to (i) bind DNA through multivalent interactions and (ii) be pH-sensitive,^37^ we expanded the scope of building blocks by introducing modified amino acids bearing an aminooxy group at the N terminus and a hydrazide at the C terminus (Fig. 1B, compounds 2).^38^ There, the presence of amino acids imparts molecular recognition properties to the system. These building blocks undergo step-growth polycondensation with a complementary synthetic bisaldehyde 3, through both acylhydrazone and oxime ligations, that lead to DCPs. Cationic amino acids (Lys, His, and Arg) were selected for the application of nucleic acid recognition, and the DCPs made of Arg gave the best results in terms of DNA complexation and siRNA delivery in live cells. Finally, our most recent achievement is the *in situ* siRNA-templated polymerization and formation of glyco-peptide DCPs capable of cell-selective siRNA delivery.^39^

### 2.3. Peptide-enforced folded DCPs

**Fig. 2:**
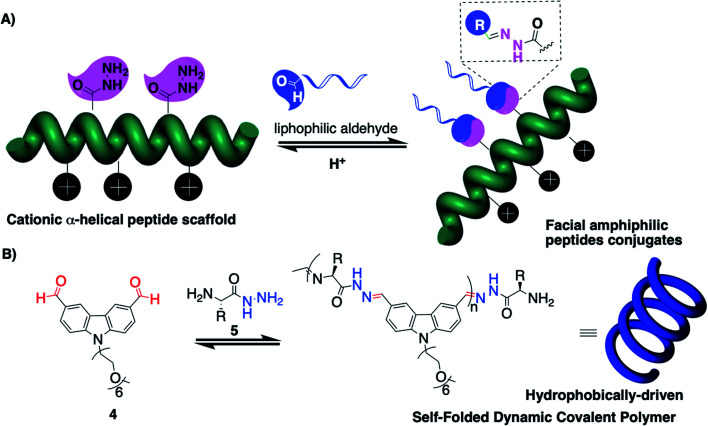
Peptide scaffolds provide facial amphiphiles in laterally-modified dynamic covalent polymers (A) and self-folded main-chain dynamic covalent polymers when assisted by the hydrophobic interactions of aromatic groups (B).

The group of Montenegro explored peptides as structured scaffolds adopting a helical secondary structure for the construction of gene delivery vectors (Fig. 2A).^40–42^ This programmed folding combined with the capacity to insert modified amino acids bearing reactive hydrazides at precise positions enables generation of amphiphiles having separated lipophilic and hydrophilic faces.

On the other hand, peptides can also enforce the folding of DCPs. Following the seminal contribution of Moore and co-workers to folding-driven formation of poly-imines,^31^ the group of Lehn explored poly-acylhydrazone DCPs and demonstrated that a hydrophobically-driven folding process can result in constitutional selection and unusually long DCPs (*M*_w_ ≈ 300 kDa).^43,44^ They then combined aromatics 4 (carbazole N-functionalized with hexaethylene glycol groups for imparting water solubility) with modified amino acids 5 (C-hydrazide amino acids that can get involved in both imine and acylhydrazone reactions at, respectively, the N- and C-termini) (Fig. 2B).^45,46^ These “proteoids” form according to a nucleation–elongation polymerization^47^ and have degrees of polymerization reaching 60 and *M*_w_ ≈ 42 kDa using a tryptophan-derived amino acid building block, while much shorter DCPs are formed when using a tyrosine-derived building block, thereby pointing out the role of the hydrophobic effect that is imparted by the aromatic amino acid side-group in the polymerization process. In the end, this example nicely illustrates the mutual effect of having aromatics and peptides within a single system, by showing how dynamic covalent and supramolecular self-assemblies can influence each other (see Section 4 for further information).

## 3. Supramolecular polymers made of aromatic-peptide conjugates

### 3.1. What are supramolecular polymers?

Supramolecular polymers are macromolecular self-assemblies in which the building blocks are held together through non-covalent interactions (*e.g.* hydrogen bonds, π–π stacking, and ion pairing).^20,21,48–50^ Like DCPs, and owing to the reversibility of this weak association, supramolecular polymers are dynamic objects capable of constitutional adaptation. This reversible association of monomers endows supramolecular polymers with unique self-healing properties.^19,33^ On the other hand, the intertwining of supramolecular polymers with the realm of biomolecules can be a fruitful route toward supramolecular biomaterials.^15^ In this regard, using bioactive supramolecular polymers, the Stupp group has recently shown that the dynamics of monomer association within supramolecular polymers plays a key role in the repair of spinal cord injury.^51^

**Fig. 3:**
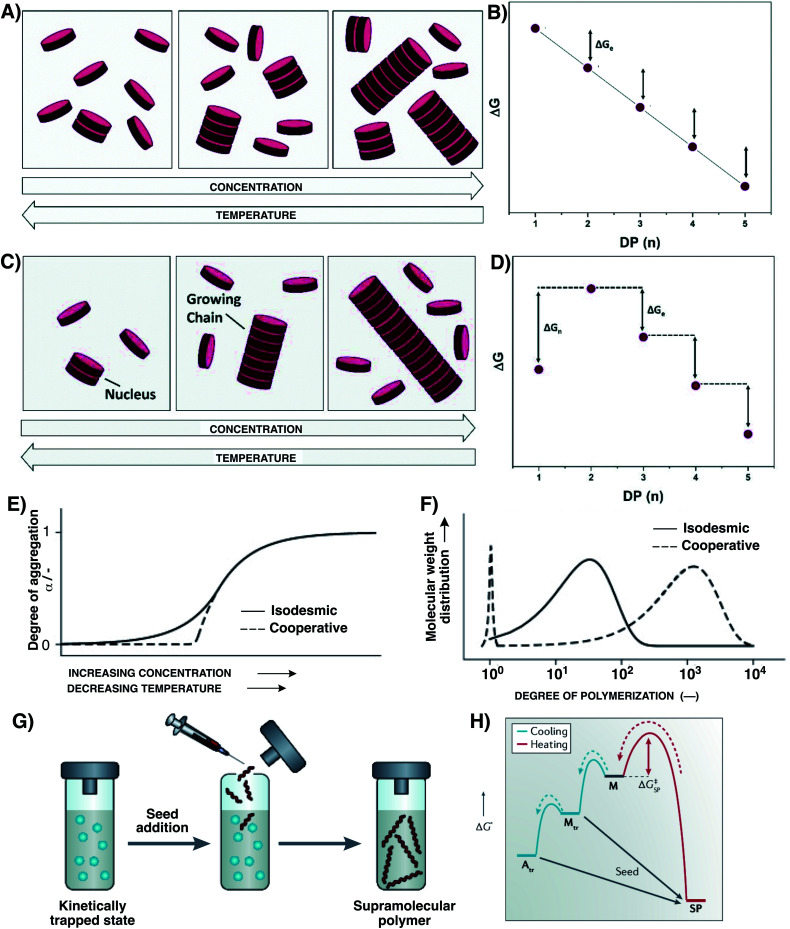
Main features of isodesmic (A and B), cooperative nucleation–elongation (C and D), and living (G and H) supramolecular polymerization processes. Panels A and C schematically depict how supramolecular polymerization is controlled by concentration and temperature, while panels B and D provide a thermodynamic view of the different steps at play with D representing the case of an uphill process involving unfavoured nuclei. Panels E and F summarize the main differences between isodesmic and cooperative polymerization processes, the isodesmic supramolecular polymerization continuously yielding small polydisperse polymers as the concentration is increased or temperature decreased, and cooperative polymerization yielding larger and less polydisperse polymers only beyond a threshold concentration and below a critical temperature. Panels G and H depict the principle of seeded supramolecular polymerization, with panel G schematically representing the process involving the addition of seeds into a solution of kinetically-trapped monomers, and panel H representing the Gibbs free energy diagram of nucleation–elongation supramolecular polymerization with the bypass involved in seeded supramolecular polymerization. Adapted from ref. 52 (permission from the Royal Society of Chemistry), ref. 55 (permission from John Wiley and Sons), and ref. 53 (copyright 2019 Springer Nature).

Supramolecular polymers can form through different mechanisms,^52–56^ and the pathway taken can have a dramatic impact on their final polymer structures. The isodesmic polymerization has been mentioned above and involves an equal association constant throughout the step-growth polymerization (Fig. 3A and B). As a result, and following Carothers' equation, this will limit the degree of polymerization and yield polydisperse supramolecular polymers. Moreover, in these supramolecular polymers that are solely under thermodynamic control, ring-chain equilibria should also be considered as a competitive path.^56^ On the other hand, cooperative polymerization can take place through a nucleation–elongation mechanism,^47,57^ characterized by a critical elongation concentration – the concentration above which elongation occurs which is inversely proportional to *K*_e_ – and a critical elongation temperature *T*_e_,^55^ which leads to a bimodal mass distribution (monomers and polymers, with little oligomer intermediates), producing longer polymers than those obtained through an isodesmic mechanism (Fig. 3C–F). In this mechanism that was first exemplified in 2006,^57^ an initial unfavourable nucleation event takes place with an association constant *K*_n_, followed by a more favourable elongation step characterized by *K*_e_ (*K*_e_ > *K*_n_). In certain cooperative systems of higher kinetic stability and displaying fast kinetics of elongation, a seeded supramolecular living polymerization gives rise to very long fibres with a very limited length dispersity (Fig. 3G and H).^58–64^ Seeding effects originate from a template-accelerated growth that occurs on small aggregate fragments – usually obtained after fragmentation (by sonication) of nano-assemblies (*e.g.* fibers and micelles)^65–67^ – which trigger supramolecular living polymerization by overriding the energy barriers (Fig. 3H). This methodology enables the programmed assembly of supramolecular block copolymers well-defined in both length and sequence (see Section 5.1).^68^ Recent endeavours have reported the photo-control of living polymerization in 2 dimensions.^69,70^

A vast category of supramolecular polymers exploits the self-assembly of aromatics through π–π stacking that mainly relies on dispersion interactions.^71,72^ However, only in cases where additional dipole–dipole interactions take place, a cooperative mechanism can emerge that requires long-range attraction forces (typically electrostatics arising from the creation of a macrodipole in the supramolecular polymer like in benzene-1,3,5-triscarboxamides, *vide infra*).^54^ Thus, the combination of aromatics with peptides capable of partaking in hydrogen bonds represents an interesting approach.

### 3.2. Effect of aromatics on peptide self-assembly

**Fig. 4:**
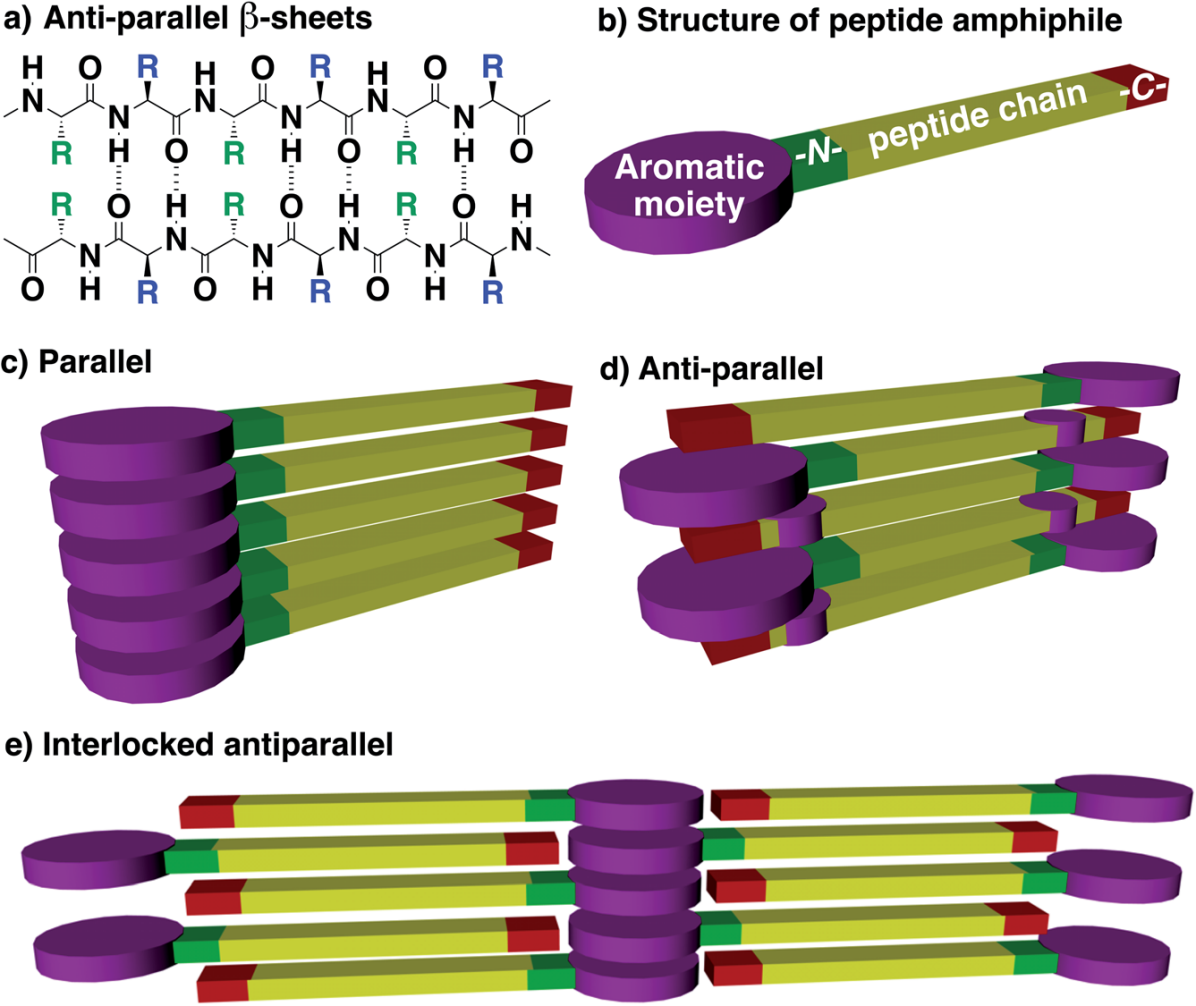
Supramolecular self-assembly of aromatic peptides: (a) general structure of anti-parallel β-sheets; (b) schematic representation of aromatic peptides with the aromatic group tethered to the N-terminal of the peptide chain; possible arrangements of aromatic peptide self-assemblies: (c) parallel, (d) anti-parallel, and (e) interlocked anti-parallel.

Inspired by the self-assembly of amyloid fibres, a pioneering input from the Gazit group was that a short diphenylalanine can undergo spontaneous self-assembly into tubular^73^ and spherical^74^ nanostructures in aqueous media.^75,76^ The group further studied derivatives featuring N-acetyl, C-amide, N-Boc, N-Cbz, and N-Fmoc modifications and observed marked structural differences in the resulting tubular nano-assemblies. Along with contributions from other groups, crystallographic analyses reveal that the presence of aromatic groups influences the organization of the self-assembled material in the solid state, while the unmodified PhePhe dipeptide gives discrete, hollow, and well-ordered nanotubes,^77^ extending the repeat to the tripeptide PhePhePhe resulting in planar nanostructures through β-sheet formation,^78^ and replacing phenylalanine with tyrosine yields microspheres instead.^79^ Adding a Fmoc group at the N-terminus of PhePhe reinforces π–π interactions and yields interlocked anti-parallel β-sheets,^80^ contrary to the acetyl group which leads to a canonical β-sheet structure when placed at the N-terminus (Ac–PhePhe–NH_2_).^81^ Thus, in addition to providing an extra-stabilization in proteins,^82^ π–π interactions also play a role in directing the self-assembly of short peptides, as also observed in the early 2000 by the Xu^83,84^ group.^85^ Indeed, when aromatics are present, very short peptides of 2–3 amino acids can surprisingly lead to the formation of materials such as hydrogels, thereby illustrating their contribution to stabilizing peptide-based self-assemblies.^86,87^ The properties of these materials depend on the length, composition, and sequence of peptides,^86^ but are also strongly affected by the chirality of the amino acids and by the nature of the aromatic groups.^88–96^ Such supramolecular self-assembly involves β-sheet formation, and different types of arrangement are proposed: parallel, anti-parallel or interlocked anti-parallel (Fig. 4).^97^ However, one caveat to make here for systems combining aromatics and peptides is that the distance between aromatics involved in π–π stacking interactions (3.4 Å) is slightly shorter than the distance separating two peptide strands involved in a β-sheet (≈4.8 Å). Therefore, the combination of both interactions requires some structural distortion. Leaders in this area have already reviewed in detail the effect of various aromatic groups (Fmoc,^98^ phenyl, naphthalene, pyrene, *etc.*) on the self-assembly of short peptide amphiphiles, and readers are referred to those accounts for comprehensive details and design guidelines that have emerged over the course of these studies.^99–103^

**Fig. 5:**
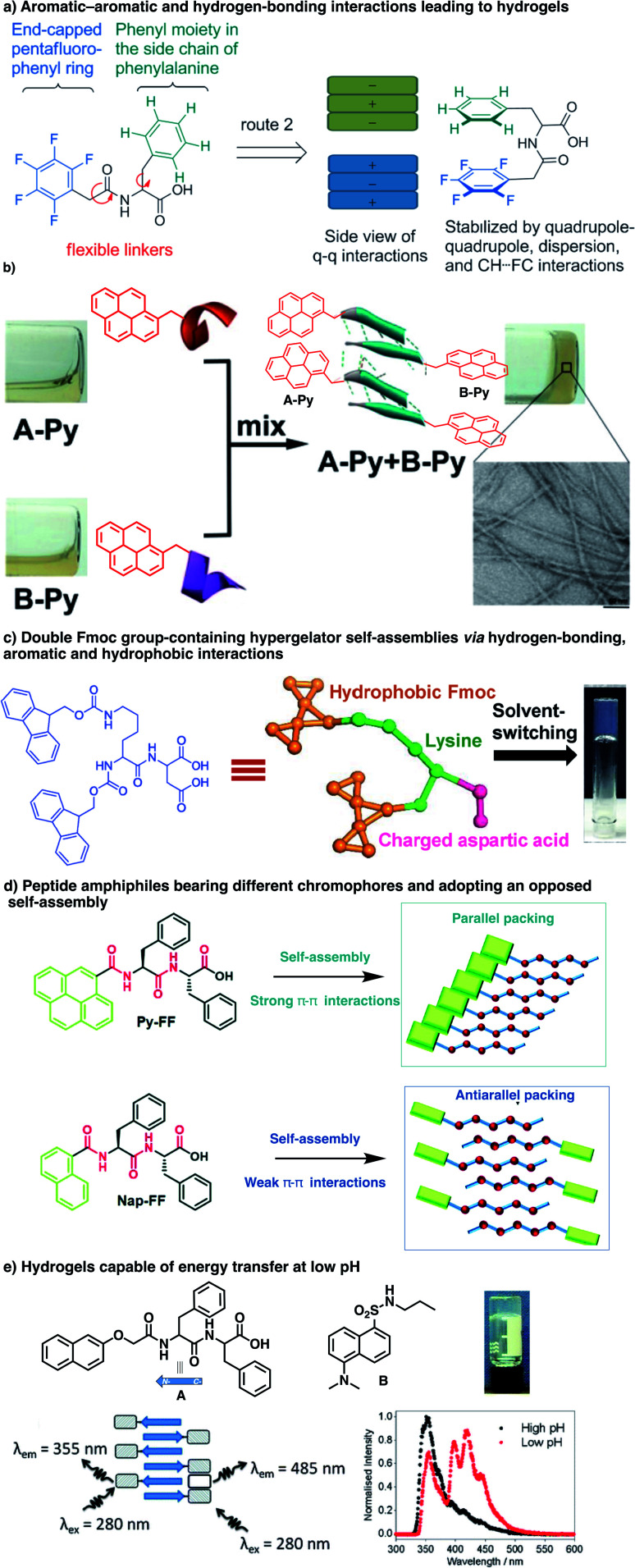
Recent examples of supramolecular polymers made of aromatic peptides: (a) hydrogel formation driven by aromatic interactions involving a penta-fluorinated phenyl as N-caps (reproduced from ref. 104 with permission from John Wiley and Sons); (b) pyrene groups trigger the self-assembly of complementary peptides in β-sheets (adapted with permission from ref. 85 copyright 2017 American Chemical Society); (c) self-complementary Fmoc-Lys(Fmoc)-Asp forms fibres and hydrogels (adapted from ref. 105 with permission from John Wiley and Sons); (d) the presence of aromatics affects the mode of assembly of PhePhe dipeptides (adapted from ref. 106 with permission from the Royal Society of Chemistry); (e) self-assembly of aromatic peptides involving complementary donor–acceptor aromatics (adapted from ref. 107 with permission from the Royal Society of Chemistry).

Aromatics are usually positioned at the N-terminus of peptides, and various structural parameters such as the nature of aromatics, structure of spacers, and composition and sequence of peptides were explored. Using such aromatic peptide amphiphiles, different types of nanostructures such as spheres, worms, sheets, tapes, fibre/tube nanostructures and hydrogel networks have been documented from incredibly low-molecular-weight compounds such as Fmoc-F and Fmoc-Y. The role of aromatic stacking interactions as a driving force for self-assembly has been revealed in several peptide derivatives. For instance, a penta-fluorinated phenyl at the N-terminus of dipeptides was shown to trigger hydrogel formation unlike its non-fluorinated analogue (Fig. 5a).^104^ Xu and co-workers reported an interesting example of complementary pentapeptides A-Py and B-Py that self-assemble through β-sheets in a process driven by the stacking of pyrene groups appended at their C-termini (Fig. 5b).^85^ Interestingly, this happens concomitantly in an α-helix to β-sheet transition in each peptide, shown by circular dichroism (CD) spectroscopy, which depends upon the distance between the aromatic moiety and the corresponding peptides. Last year, Gazit and co-workers reported an interesting dipeptide, Fmoc–Lys(Fmoc)–Asp, bearing two Fmoc groups on the N-terminal Lys, and two hydrophilic carboxylic acid moieties at the C-terminal Asp,^105^ which self-assembles in parallel stacks into fibres that lead to a hydrogel at a very low critical gelation concentration (CGC) of 0.002 wt% (Fig. 5c). Very recently, aromatics have also been shown to influence the self-assembly of PhePhe – a well-studied bioinspired prototype of soft materials (Fig. 5d).^106^ In this work, the Fernández group functionalized the N terminus of PhePhe with pyrene of naphthalene groups and found that these aromatics altered the mechanism of self-assembly. While the pyrene peptide conjugate Py-FF arranged in parallel β sheets that are enforced by strong π–π interactions, the naphthalene conjugate Nap-FF adopted an antiparallel β sheet organization in H_2_O/THF 9/1.

These organizations resulted in, respectively, aggregation-caused quenching and aggregation-induced emission of the fluorescence of thoee conjugates. Interestingly, the self-assembly pathway revealed by variable-temperature studies of UV-Vis spectroscopic data is a highly cooperative (*σ* = 4.5 10^−5^, see below) supramolecular polymerization for the π-stacked pyrene conjugate and an isodesmic self-assembly for the naphthalene peptide conjugate. Complementary aromatics displaying charge transfer interactions can also be used for the formation of multi-component systems (Fig. 5e).^107^ Finally, manipulating the extent of π–π/hydrophobic interactions involving aromatics should then enable modulation of the thermodynamic stability of the resulting nanostructure. Indeed, the photo-switching of azobenzenes was shown to enable control of gel–sol transition with light.^108–110^

**Fig. 6:**
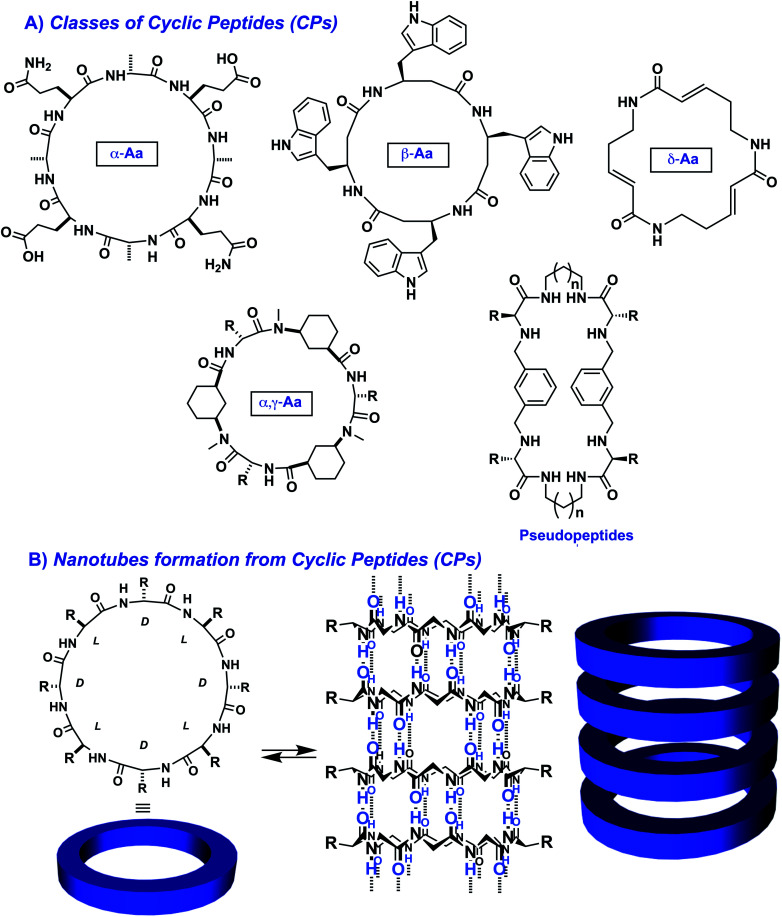
Examples of cyclic peptides (CPs) used for generating nanotubes through supramolecular polymerization: (A) representative structures of cyclic peptides made of α-*alt*(l,d) residues,^112^ β-residues,^113^ δ-residues,^114^ α- and γ-residues,^115^ and cyclic pseudopeptides;^116^ (B) self-assembly of cyclic peptides through β-sheet formation leading to nanotubes.

Cyclic peptides (CPs) form a particular class of compounds capable of self-assembling into columnar nanostructures. These peptides can be obtained by an amide bond cyclization between the N- and the C-termini of a peptide chain (Fig. 6).^111^ Within these CPs, the amide groups are perpendicularly arranged to the plane of the ring, thereby promoting the formation of hydrogen bonds with other cyclic peptides *via* tubular-assembly. As a result, nanotubes are generated, in which the inner diameter and properties depend on the number and nature of the building blocks.

Initially pioneered by the group of Ghadiri,^112^ the Granja and Montenegro labs have provided a seminal contribution in the last decade to the synthesis and self-assembly of octameric d/l-alternating cyclic peptides. In particular, they pioneered the use of cyclic γ-amino acids (γ-Aca) in combination with natural α-amino acids of opposite chirality. This combination produces peptides that tend to acquire a flat conformation, which facilitates their supramolecular polymerization. Consequently, long hollow tubular structures are obtained through the 1D columnar assembly of CPs.^117–120^

In order to control the longitudinal growth of the nanotubes, Perrier and co-workers showed the prominent effect of polymers as side-chains of CPs which limit 2D and 3D assemblies and reinforce the non-covalent associative interactions between CPs by shielding the core from the surrounding water.^121–126^

**Fig. 7:**
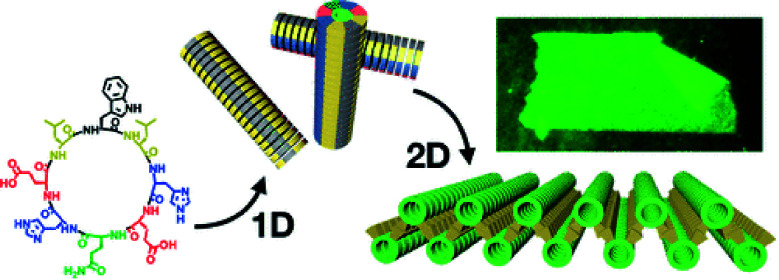
Two-dimensional self-assembly of d/l-alternating cyclic peptides by sequential 1D-to-2D supra-molecular polymerization through leucine zippers and π–π stacks of tryptophan indoles. Reprinted with permission from ref. 132. Copyright 2020 American Chemical Society.

On the other hand, hydrophobic and aromatic amino acids, well positioned in those cyclic peptides, also participate in their supramolecular polymerization. For instance, pyrenes were reported to promote the hierarchical supramolecular polymerization of CPs,^127,128^ favouring fibres and bundles due to the mismatch between the β-sheet and π–π stacking that results in interlocking of fibres into bundles.^129^ Controlling the balance of attractive and repulsive forces could grant control over the supramolecular polymerization of CPs. An example was recently reported where a spiropyran photo-switch had been inserted into CPs and afforded fast supramolecular polymerization and depolymerization in response to light irradiation triggering pH changes which modulate coulombic interactions.^130^ Finally, the presence of hydrophobic and aromatic amino acids can be very useful for controlling the 2D growth of CPs. For instance, CPs bearing leucine, phenylalanine and tryptophan in key positions lead to the formation of giant 2D-nanosheets by involving leucine zippers and aromatic stacks in aqueous media (Fig. 7).^131,132^

A particular class of peptide conjugates that has been extensively studied for their self-assembly propensity is amphiphilic peptides. These peptides are modified with hydrophobic moieties, most often aliphatic chains placed at their N-terminus.^133^ The group of Stupp reported numerous pioneering examples of spherical and cylindrical micelles or lamellar structures.^133–135^ Like α-helical peptides,^136^ amphiphilic aromatic β-sheet-forming Glu–(Phe–Glu)_*n*_ peptides are capable of self-replication with exponential growth.^137^ Looking at synthetic aromatic-peptide conjugates, Fmoc-VFFAKK is also capable of self-replication, and the kinetic control over this process enables the production of peptide nanofibers with controlled lengths and dispersity using a seeded supramolecular polymerization (Fig. 5G and H and *vide infra*), which is of great importance because length distribution determines the mechanical properties (*e.g.* stiffness) of the resulting hydrogels.^138^ Other examples using, for instance, Fmoc-GFFYGHY reported the autonomous growth of hydrogels having emerging esterase-like activity that promotes its auto-catalytic growth.^139^

### 3.3. Effect of peptides on supramolecular polymerization of aromatics

**Fig. 8:**
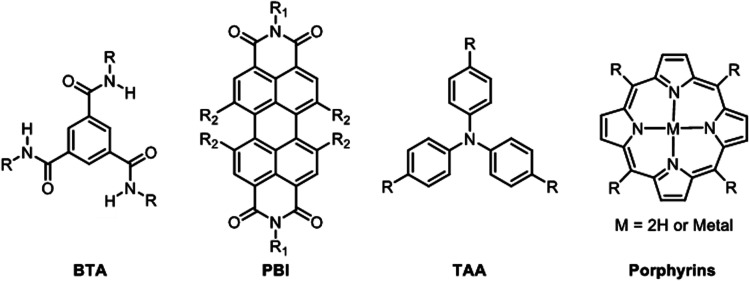
Aromatic cores most commonly used in supramolecular polymers involving poly-association by π–π stacking interactions: benzene-1,3,5-triscarboxamide (BTA), perylene-bisimide (PBI), triarylamines (TAA), and porphyrins.

Supramolecular polymers involving the self-assembly of aromatics have been a subject of intense research during the last two decades, and several compounds have emerged as leads in this area: benzene-1,3,5-triscarboxamide (BTA),^140^ perylene-bisimide (PBI),^141,142^ triarylamines (TAA),^143–145^ and porphyrins^60,64,146,147^ (Fig. 8).

The group of Meijer pioneered the use of BTAs for making supramolecular polymers. BTAs undergo a cooperative supramolecular polymerization that originates from the subtle combination of π–π stacking interactions between the aromatic cores with hydrogen bonds between the amide groups, resulting in the creation of a strong macrodipole (36 D) due to the enforced rotation out-of-the-plane (45°) of the amide groups within the final helical supramolecular polymer.^54,140^ BTAs have been extensively studied in nonpolar organic solvents, and numerous derivatives have been reported indicating that the nature of the side chains has a profound impact on the supramolecular polymerization mechanism, going from isodesmic to cooperative polymerization with a wide range of cooperativity factor (*σ* = *K*_n_/*K*_e_) ranging from 10^−1^ (weakly cooperative) to 10^−6^ (strongly cooperative).^148^ Since their inception, BTAs have been conjugated to peptides, thereby providing four main advantages that are discussed hereafter: (i) improving the water-solubility of supramolecular polymers which is a daunting challenge for aromatic-based supramolecular polymers,^149^ (ii) adding additional non-covalent interactions that may affect the polymerization mechanism, (iii) inserting amino acids bringing molecular chirality information that can be transmitted to the supramolecular organization,^150^ and (iv) introducing functional groups within the final supramolecular polymers in a straightforward and versatile manner.^16,151^

#### 3.3.1 Inserting peptides to improve water-solubility

**Fig. 9:**
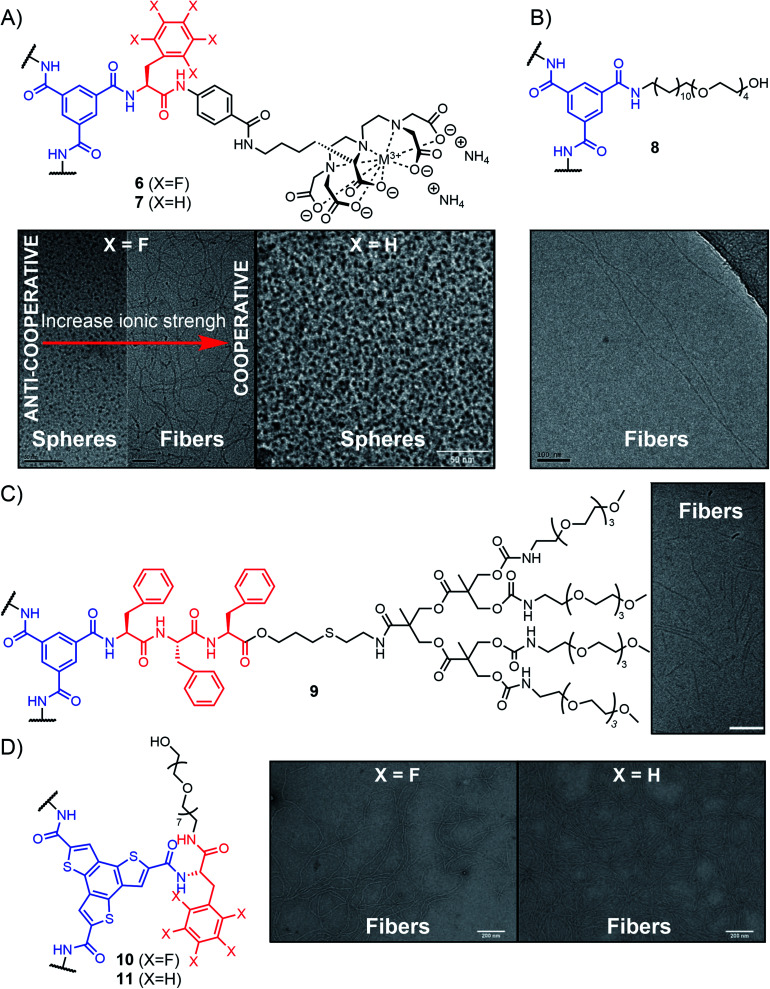
Aromatic-peptide conjugates self-assembling into supramolecular polymers in aqueous media with representative characterization of nanostructures observed by TEM. (A) Adapted from ref. 152 with permission from John Wiley and Sons and ref. 153 (copyright 2010 National Academy of Sciences); (B) adapted from ref. 154 with permission from the Royal Society of Chemistry; (C) adapted from ref. 155 with permission from John Wiley and Sons; (D) adapted from ref. 159 with permission from the Royal Society of Chemistry.

Meijer and co-workers reported in 2010 that flanking the central BTA unit with highly charged peripheral groups – made of penta-fluorinated l-phenylalanine, an aminobenzoate spacer, and a polycarboxylate ligand complexed to Gd^III^ – enables water-solubility to be achieved (Fig. 9A, compound 6).^152,153^ The system displays a combination of attractive forces (hydrogen bonding, π–π interactions, and the hydrophobic effect), along with repulsive electrostatic interactions that frustrate the one-dimensional growth of supramolecular polymers. The polycarboxylate ligand displays a varying number of negative charges which enable fine-tuning of the repulsive forces. The coulombic repulsion was shown to affect the morphologies of the resulting nanostructures as well as the mechanism of supramolecular polymerization. While high aspect ratio rod-like aggregates are obtained through cooperative polymerization in high ionic strength aqueous media, discrete spherical objects were formed when coulombic repulsion dominates under low ionic strength conditions (Fig. 9A). The length and the rigidity of the hydrophobic peptides were found to be critical – only spheres being formed when penta-fluorinated phenylalanine was replaced by phenylalanine (Fig. 9A, compound 7). Other examples of BTAs provided by the same group using aliphatic and PEG side-chains have further demonstrated the role of hydrophobic shielding in cooperative supramolecular polymerization in water, leading to rod-like self-assemblies with nanometer diameters and micrometer lengths (Fig. 9B, compound 8).^154^

Besenius and co-workers followed a different design to achieve supramolecular polymerization in aqueous solutions. They used BTAs conjugated to amphiphilic peptides forming β-sheets, end-capped at their C-terminus with a tetra(ethylene glycol) dendron for water solubility (Fig. 9C, compound 9). Using a triphenylalanine peptide yields nanorods that are formed through a non-cooperative isodesmic polymerization originating from the steric constraints that grow throughout the polymerization (frustrated growth).^155^ The group also developed alternate supramolecular polymers by using complementary peptides of opposite charges, rich in glutamic and lysine amino acids, respectively. At neutral pH, the two peptides interact with each other forming a β-sheet structure reinforced by favourable electrostatic interactions, while at acidic or basic pH, only one species is in a non-ionic form, leading to the formation of homopolymers instead of the alternate supramolecular polymers.^156–158^

Other examples have shown that appending peptides can impart water solubility to various aromatic cores that would otherwise not be amenable to supramolecular polymerization in aqueous media. For instance, using C3-symmetric benzotrithiophene functionalized with l-phenylalanine peptides, García-Iglesias and co-workers obtained supramolecular fibres that are self-assembled in water through a delicate combination of hydrogen bonding and hydrophobic effects (Fig. 9D, compounds 10 and 11).^159^

#### 3.3.2 Effect of peptides on the mechanism of supramolecular polymerization

**Fig. 10:**
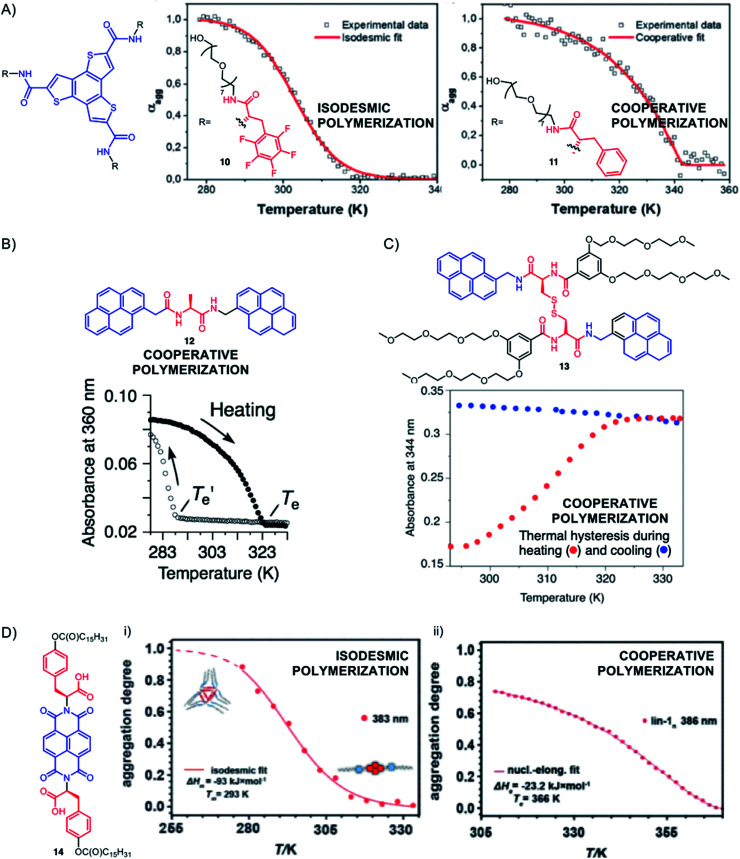
Selected examples showing how peptides can affect the mechanism of supramolecular polymerization. The depicted plots represent the evolution of the degree of aggregation *α*_agg_ in (A) and (D), or the evolution of the absorbance in (B) and (C), as a function of temperature – the sigmoidal or non-sigmoidal changes revealing, respectively, isodesmic or cooperative supramolecular polymerization mechanisms. (A) Adapted from ref. 159 with permission from the Royal Society of Chemistry; (B) reproduced from ref. 160 with permission from John Wiley and Sons; (C) adapted from ref. 161 with permission from the Royal Society of Chemistry; (D) adapted from ref. 162.

The presence of peptides can have a tremendous effect on the mechanism of supramolecular polymerization as a function of the nature of (aromatic) side-groups. For instance, in the benzotrithiophene peptide conjugates reported by García-Iglesias and co-workers, the authors found that the pentafluoro-l-phenylalanine side-chain in compound 10 induces an isodesmic self-assembly process dominated by hydrophobic forces, while the l-phenylalanine side-chain in compound 11 gives rise to a highly cooperative supramolecular polymerization (*σ* ≈ 8 10^−6^) thanks to a greater contribution of the hydrogen bond (Fig. 10A).^159^ Yamaguchi and co-workers reported that compound 12 featuring two pyrenes linked through an alanine-based diamide motif can exist either in a dominant folded state involving intramolecular hydrogen bonds or in an open state involving intermolecular hydrogen bonds and that only the latter can give rise to cooperative supramolecular polymerization in low polarity solvents (toluene/chloroform) (Fig. 10B).^160^ The retardation of spontaneous nucleation in the folded state enables seeding to trigger an out-of-equilibrium polymerization. More recently, the group has extended their approach studying the cystine-based dimeric diamide 13, which now shows seeded polymerization in aqueous media (Fig. 10C).^161^

The Smulders and Stefankiewicz groups used a naphthalene diimide (NDI) flanked with two O-derivatives of l-tyrosine (l-Tyr(OC(O)C_15_H_31_)COOH) to generate soluble self-assemblies in non-polar organic solvents (CHCl_3_ and MCH) and interestingly found that the solvent affects both the type of objects formed (hydrogen-bonded nanotubes *vs.* supramolecular polymers) and their mechanisms of formation (isodesmic or cooperative) (Fig. 10D, compound 14).^162,163^ These subtle changes are rationalized by differences in solvation effects between J-aggregates (offset face-to-face arrangement of aromatics) and H-aggregates (overlapped arrangement of aromatics). Such differences in competitive pathways of supramolecular polymerization involving either J- or H-aggregates have also been documented in other systems.^164,165^ Other examples of NDI–peptide conjugates have been reported for supramolecular polymerization of 1D helical nanostructures in aqueous media^166^ and nanotubes,^167^ some assemblies even displaying pH-responsiveness due to the presence of ionizable amino acids.^168^

#### 3.3.3 Chiral supramolecular polymers

**Fig. 11:**
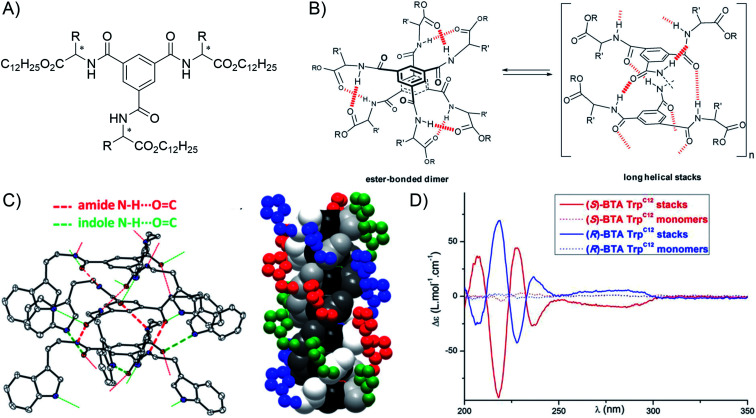
Example of chiral supramolecular polymers: (A) general structure of BTA-amino acid esters (dodecyl esters shown here); (B) representation of the equilibrium between dimers and long helical supramolecular polymers (reproduced from ref. 169 with permission from the Royal Society of Chemistry); (C) X-ray structure of the (*S*) tryptophan isopropyl BTA with the ORTEP representation of a trimer showing the two types of hydrogen bonds present in the helical polymer (left), and a diagram of the hexamer highlighting the inner helical threefold hydrogen bond network (atoms in different shades of grey, and the BTA ring in black) surrounded by the second helical network involving the indole moiety (atoms in color) (right). The ester side-chains (CO^i-Pr^_2_) and hydrogen atoms are omitted for clarity (reproduced from ref. 172 with permission from the Royal Society of Chemistry); (D) CD spectra of monomers and supramolecular polymers made from tryptophan dodecyl BTA of different chirality (reproduced from ref. 172 with permission from the Royal Society of Chemistry).

The group of Bouteiller has been particularly active in the investigation of BTA–amino acid conjugates (Fig. 11A). In 2015, they reported that the presence of α-amino dodecylester side groups (methionine, norleucine and phenylalanine dodecyl ester) favors the formation of long rods at millimolar concentrations in cyclohexane,^169^ and displays a strong chiral amplification effect in the helical nanostructure, as seen by CD spectroscopy, through the so-called “sergeants-and-soldiers effect” that operates in copolymers made of a mixture of chiral and achiral monomers.^169,170^ Further work on BTA derivatives with valine dodecyl ester revealed a propensity of heterochiral monomers to form long rods while homochiral analogues only formed dimers (Fig. 11B), which can be detected by increases in relative viscosity.^171^ On the other hand, tryptophan alkyl ester side groups were found to promote supramolecular polymerization through the presence of additional hydrogen bonds involving indole N–H (Fig. 11C).^172^ Small-angle neutron scattering experiments revealed long and rigid one-dimensional cylinders with *L* > 1000 Å and DP_w_ > 275, and circular dichroism showed intense signals that originate from the chiral nanoscale organization of the supramolecular polymers (Fig. 11D).

#### 3.3.4 Inserting peptides for achieving functional systems

To illustrate some applications of supramolecular polymers based on aromatic peptide conjugates, we mention hereafter three potential outcomes that have been recently reported.

**Fig. 12:**
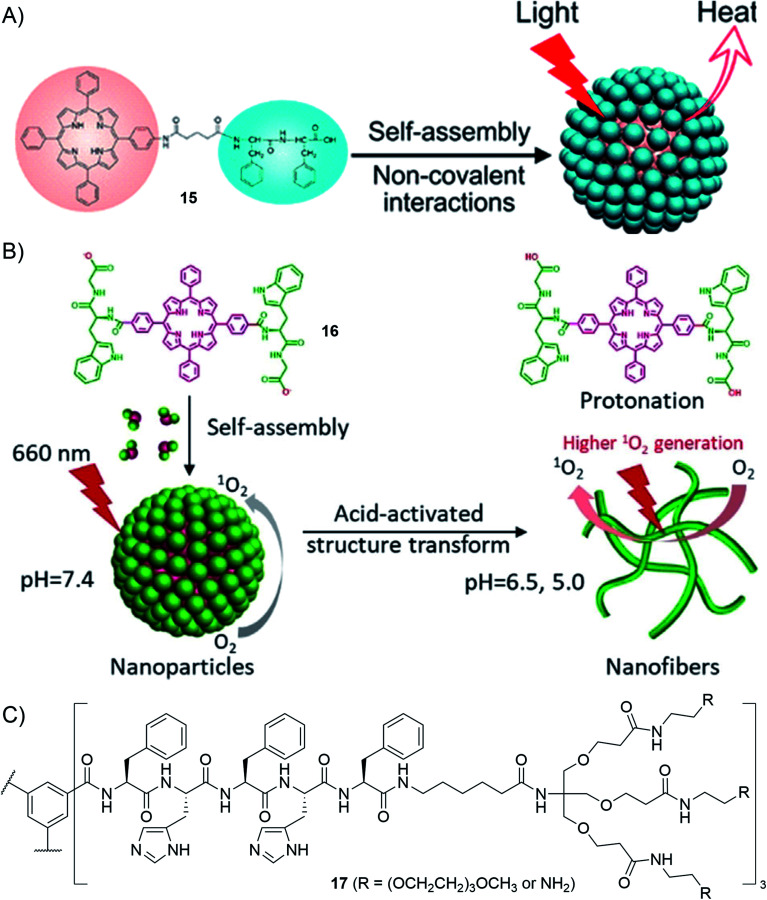
Selected examples of applications using supramolecular polymers decorated with peptides: (A) porphyrin–peptide conjugates for photothermal therapy (reprinted with permission from ref. 173. Copyright 2017 American Chemical Society); (B) acid-activatable porphyrin–peptide conjugates for photodynamic therapy (adapted from ref. 175 with permission from John Wiley and Sons); (C) molecular structure of the BTA-derived monomer leading to pH-switchable supramolecular polymers for siRNA delivery.^176^

First, conjugation with peptides enables improvement of the water-solubility of aromatic photosensitizers whose biological applications are otherwise often hindered by their poor solubility in aqueous media. For instance, porphyrin–peptide (PhePhe) conjugates 15 having a short spacer have been reported to self-assemble into nanodots in aqueous media which, thanks to the peptide-enforced π-stacked arrangement of chromophores, are active in photothermal therapy (Fig. 12A).^173^ The presence of PhePhe, on porphyrin–peptide conjugates with no spacers, was shown to profoundly impact the morphology of the nanostructures that are formed, yielding fibrils, platelets or nanospheres depending on the solvent composition.^174^ The control over the nano-scale organization has been recently exploited to demonstrate, using compound 16, acid-activated photodynamic therapy (PDT) that originates from an acid-triggered conversion of nanoparticles to nanofibers, the latter having greater PDT activity than the former (Fig. 12B).^175^

Secondly, the presence of peptides may also bring responsiveness to supramolecular polymers. For instance, inserting histidines within their BTA–peptide conjugates (BTA–(Phe–His–Phe–His–Phe–X)_3_), the group of Besenius was able to tune the pH-switch of supramolecular polymers, allowing control over their assembly/disassembly in the pH range 5.3–6.0 (Fig. 12C, compounds 17).^176^ Protonation of all low-pKa imidazole groups builds up electrostatic repulsion between monomers and thus triggers depolymerization. A successful application was then shown for the delivery of siRNA. In addition to the histidine-rich core, the design features peripheral primary amines which are protonated at physiological pH and are responsible for siRNA complexation. Acidification during endocytosis triggers depolymerization of the supramolecular polymer vector and thus improves siRNA release.^176^ Cationic derivatives of benzotrithiophene peptide conjugates bearing terminal primary amines have been recently described where pH was observed to cause changes, not only switching supramolecular polymerization on and off, but also affecting the mechanism of supramolecular polymerization.^177^ In this case, a highly cooperative polymerization (*σ* = 2.3 10^−5^) was observed at basic pH (pH = 13) while an isodesmic polymerization was found at lower pH (pH = 7) where amines are protonated and exert electrostatic repulsion. The nature of the aromatic core may also modulate the pH-responsiveness as recently reported by the groups of Besenius and George using different core-substituted NDI derivatives.^168^

Thirdly, the ability to self-assemble small molecules into chiral supramolecular polymers opens up applications in different areas. For instance, exploring asymmetric catalysis, the group of Bouteiller and Raynal used chiral BTA ligands for rhodium-catalyzed asymmetric hydrogenation^178^ and hydrosilylation reactions.^179,180^ Interestingly, the sergeants-and-soldiers principle enables use of a ligand-free enantiopure chiral comonomer (down to 0.5 mol%) and an achiral BTA ligand as, respectively, sergeants and soldiers, to achieve asymmetric hydrogenation with an ee up to 90%.^181,182^ This effect results from the translation of the sergeants-and-soldiers principle to asymmetric catalysis through chiral amplification that originates from the self-assembly of monomers into helical supramolecular (co)-polymers.^181,183^ The ability to self-organize aromatics into chiral aggregates may also open applications in the field of organic electronics.^184^ In this case, the group of Frauenrath combined peptides with polymer side-chains to assemble perylene bisimide, quaterthiophene and other chromophores into chiral nanowires.^185–187^

## 4. Hierarchical self-assembly of aromatic-peptide conjugates through the combination of dynamic covalent and supramolecular chemistries

In retrospect, Rideout provided in 1986 a pioneering example of a self-assembling drug where dynamic covalent bond formation subsequently triggers supramolecular self-assembly in a hierarchical two-step process.^188^ In 2010, Ulijn has nicely reviewed the then-growing interest in merging dynamic covalent chemistry and peptide self-assembly.^189^ To illustrate recent trends with selected examples, Montenegro and co-workers described the hierarchical self-assembly of micro-fibres from linear peptide amphiphiles using oxime ligation^190,191^ and a single covalent connector between a charged peptide head and a lipophilic aliphatic tail,^192,193^ while Jierry and co-workers reported the protein-induced low-molecular-weight hydrogelator formation through templated ligation of peptides by a disulphide link.^194^ In 2020, George and co-workers reported a very elegant chemically-controlled supramolecular polymerization where a dynamic covalent bond (imine) converts a non-assembling dormant charge-transfer monomer to an activated amphiphile that subsequently undergoes a cooperative supramolecular polymerization.^195^ The Ng and Weil groups reported an elaborate system where a pH-sensitive reversible linkage is connected to a precursor of a self-assembling peptide.^196^ The authors showed that the dissociation of the covalent bond at acidic pH triggers the release of the peptide and the formation of self-assembled fibres within live cells.

**Fig. 13:**
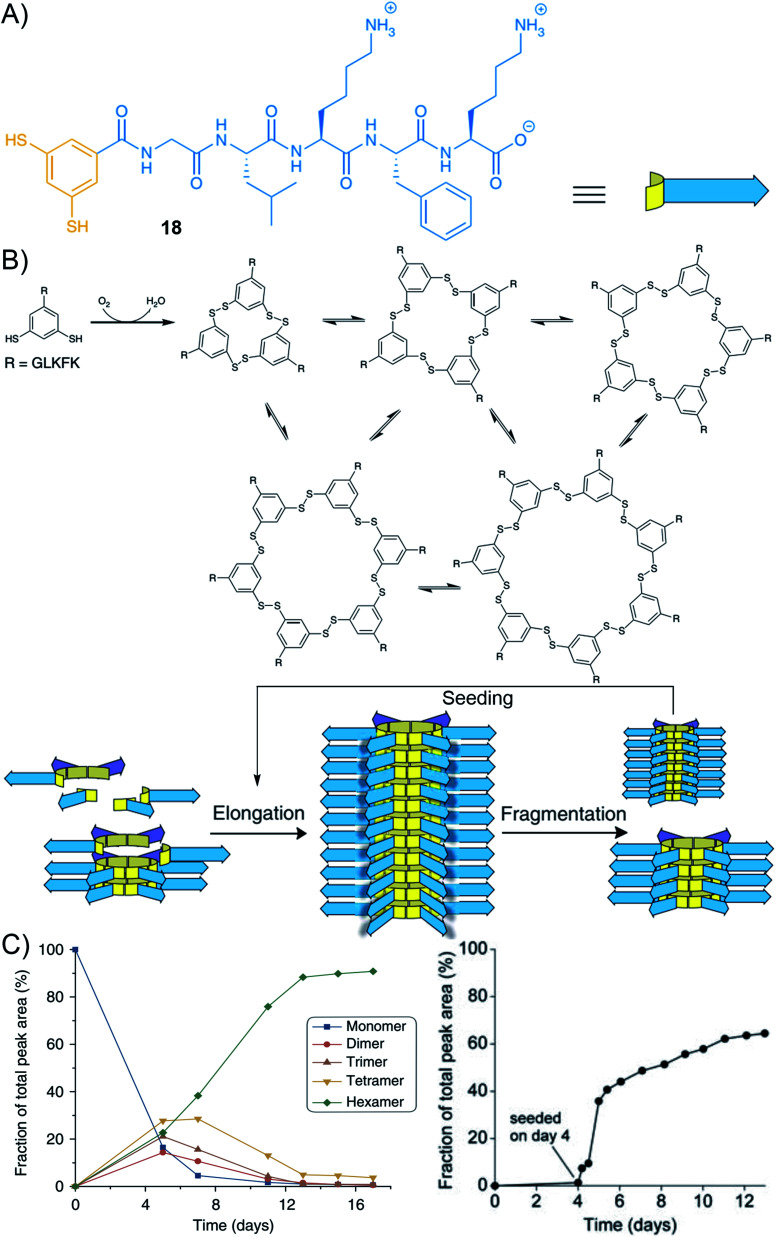
Hierarchical self-organization of aromatic peptide conjugates through disulphide dynamic covalent self-assembly and supramolecular polymerization: (A) molecular structure of building block 18 made of the peptide Gly–Leu–Lys–Phe–Lys tethered to the 1,3-dimercaptobenzene aromatic core; (B) schematic representation of its self-assembly at the molecular (top) and supramolecular (bottom) scales upon air oxidation, forming different macrocycles that can undergo supramolecular polymerization; (C) representation of the evolution of the constitution of the system showing the amplification of the hexamer (left) and its templated growth in the presence of seeds (right). Adapted from ref. 198 (copyright 2010, American Association for the Advancement of Science) and ref. 204.

Beside the hierarchical generation of complex nanostructures from two complementary simple building blocks, there is also a strong interest in understanding the pathways that are taken by more complex systems characterized by the integration of multiple (>2) building blocks.^197^ In this regard, there have been many beautiful results, in the last decade, of systems combining aromatics with peptide self-assembly. In 2010, while studying artificial self-replicating molecules, the group of Otto came across mechano-sensitive self-replication driven by a complex self-organization process combining covalent self-assembly with supramolecular polymerization.^198^ The design is based on aromatic-peptide conjugates where the aromatic bears two *meta*-oriented thiol groups that can further lead to disulphide bond formation and the peptide features alternating hydrophobic and hydrophilic amino acids (Gly–Leu–Lys–Leu–Lys–OH or Gly–Leu–Lys–Phe–Lys–OH) (Fig. 13, compound 18). The authors found that the corresponding hexamer and heptamer are formed through a sigmoidal growth that is triggered by mechanical forces. Seeding experiments demonstrated self-replication, yielding long fibres of micrometre size. Further analyses by CD spectroscopy and thioflavin T assay confirmed the β-sheet arrangement of the peptides. Overall, these results are interpreted by the disulphide-mediated covalent assembly of aromatic-peptide conjugates that produce rings of different sizes, from which one is prone to nucleation–elongation supramolecular polymerization by π–π stacking and β-sheet formation and emerges by exponential self-replication (Fig. 13).^199–204^ With the assistance of an appropriate template, it is also possible to down-size the building block to a minimal size that now includes a “peptide” side-group as short as Phe–OH.^205^ Finally, in a recent study in collaboration with the Huc group, it was shown that low-symmetry foldamers spontaneously emerge from the system when amphiphilic peptides are tethered to the 1,3-dimercaptobenzene aromatic core, thereby documenting a beautiful example of complex self-organization into discrete macromolecules (Fig. 2C).^206^

**Fig. 14:**
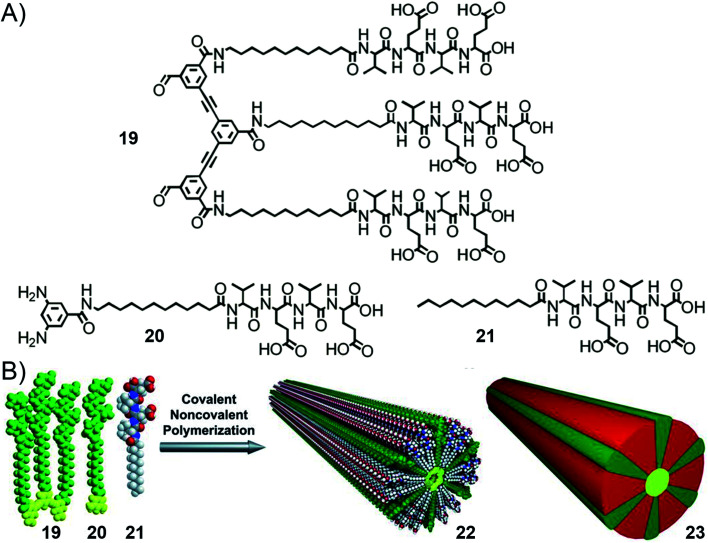
Hierarchical self-organization of amphiphilic aromatic peptide conjugates through imine dynamic covalent self-assembly and supramolecular polymerization: (A) molecular structures of the bisaldehyde (19), bisamine (20) and peptide amphiphile (21) building blocks; (B) schematic representation of the synergistic process involving the hybrid covalent-supramolecular polymerization of a macrocyclic rosette by mixing monomers 19, 20, and 21 in a 1 : 1 : 2 molar ratio in aqueous solution (pH 5), yielding the 1D fiber assembly 22. Schematic representation 23 depicts the hybrid covalent-noncovalent polymer with different colors for the covalent (green), dynamic covalent (yellow) and supramolecular (red) interactions at play. Adapted with permission from ref. 207 (copyright 2016, American Association for the Advancement of Science).

In 2016, the group of Stupp reported another system of supramolecular polymerization achieved through simultaneous hierarchical covalent and non-covalent self-assembly.^207^ The building blocks are also made of aromatic peptide conjugates but the aromatic bears either two reactive aldehyde or amine groups, and the peptides (Val–Glu–Val–Glu–OH) are separated by C_11_-alkyl spacers (Fig. 14). Mixing of two complementary building blocks (*i.e.* bisaldehyde and bisamine) 19 and 20 in a 1 : 1 molar ratio in aqueous solution at pH = 5 triggers imine formation, and the authors observed by cryo-TEM the heterogeneous mixture of one-dimensional structures. In contrast, with further addition of 2 equivalents of peptide amphiphile 21 (dodecyl–Val–Glu–Val–Glu–OH), a hybrid covalent-noncovalent polymer is generated, displaying 1D structures with a precisely defined cylindrical shape and uniform diameter. β-sheet organization of the peptides was evidenced by CD spectroscopy. These results describe an interesting case where covalent and non-covalent processes occur simultaneously and synergistically to trigger the formation of polymers (up to an average molecular weight of 190–250 kDa) following a nucleation–elongation mechanism, which is much higher than for analogous covalent-only polymers having average molecular weights on the order of 14 kDa.^133^

**Fig. 15:**
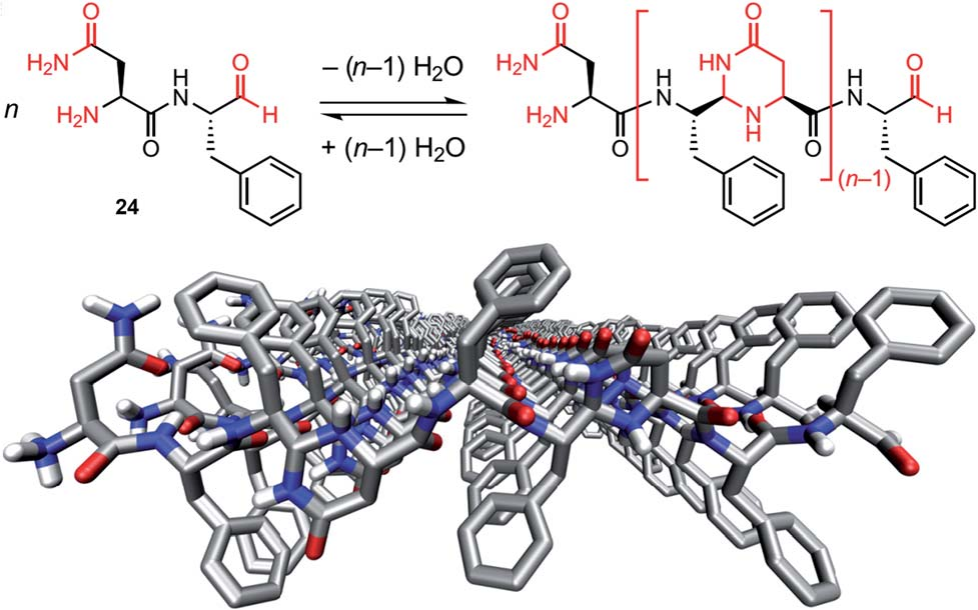
Dynamic covalent polymers that further self-assemble in β-sheet arrangements through supramolecular polymerization. Top: principle of self-condensation polymerization of peptide 24 (H–Asn–Phe–CHO) that yields oligomers upon formation of *N*,*N*-acetal-4-pyrimidinone (py) linkages; bottom: model of the β-sheet assembly of the trimer H–NFFpyNFFpyNFF–CHO generated from H–Asn–Phe–Phe–CHO. Reproduced from ref. 208 (copyright 2017 Springer Nature).

In 2017, the group of Lynn reported modified peptides 24 that undergo both dynamic covalent and supramolecular polymerization driven by β phase formation.^208^ The system is designed around the (Phe)_1–2_ core with a C-terminal aldehyde and a complementary N-terminal asparagine, and yields mixtures of cyclic and linear oligomers by poly-condensation in acidic acetonitrile, before the appearance of a particle phase and formation of fibres (Fig. 15). The authors evidenced self-selection and amplification of trimers as a consequence of the emergence of these fibres,^208,209^ thereby showing an example of adaptive self-assembly with feedback loops linking the emergence and growth of the nanostructure with the selection of dynamic covalent polymers.

**Fig. 16:**
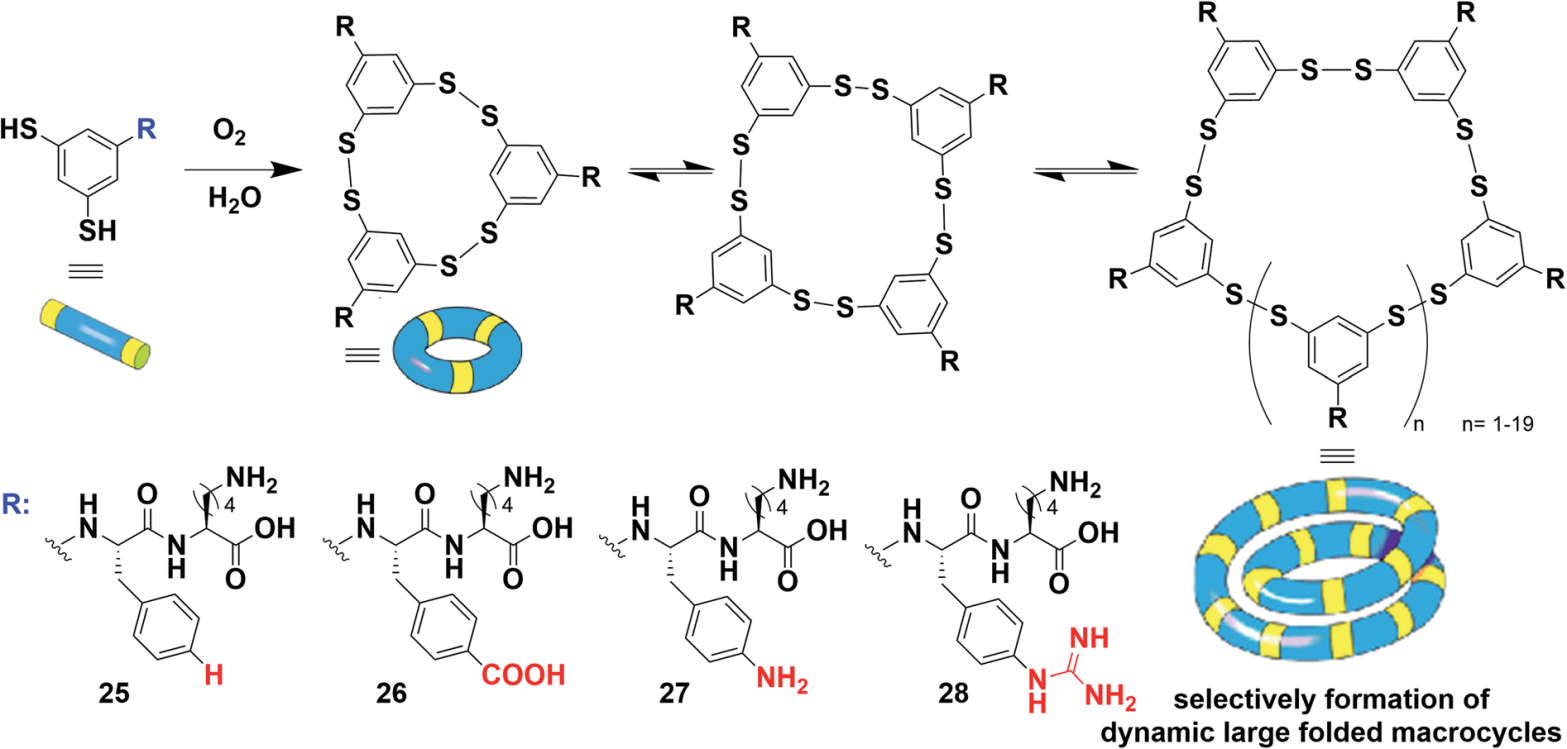
Large folded monodisperse macrocycles formed through hierarchical self-assembly combining dynamic covalent chemistry with supramolecular self-assembly of aromatic peptide conjugates. Adapted from ref. 206 (copyright 2020 Springer Nature).

Instead of folding into a repetitive helical polydisperse nanostructure, the groups of Huc and Otto have recently reported a system where the presence of amphiphilic peptides drives the protein-like folding of the system into a low-symmetry monodisperse object (Fig. 16).^206^ In this case, the presence of charged groups on the phenyl ring of peptide side groups was found to be critical. In the absence of these charged groups, only tetramers formed from compound 25. On the other hand, the self-assembly of 26 resulted in mixtures of 9-, 12-, and 13-mers, and 27 and 28 spontaneously yielded, respectively, 23- and 16-mers.

Finally, it is important to note that the combination of dynamic covalent and supramolecular chemistries also brings an additional covalent responsiveness to the system as a result of different reaction conditions. The reversible covalent bonds are sensitive to reaction conditions and thus, in addition to the sensitivity of non-covalent interactions to various modulators such as solvent and temperature, such a double-dynamic system may display responsiveness to multiple stimuli. For instance, the systems made of disulphides are redox sensitive while those featuring imines or acylhydrazones may be photo-sensitive since those motifs can be photo-switched.^210,211^ In this line, the Hamachi group recently reported the formation of photo-triggered out-of-equilibrium patterns in hydrogels using photo-responsive nanofibers made of aromatic-peptide acylhydrazone conjugates.^212^

## 5. Recent developments

### 5.1. Multi-component precision polymers

Despite tremendous recent progress,^213,214^ synthetic polymers with precise sequence-defined positioning of multiple monomers remain far out of reach. Yet, such objects are of great interest as they can be seen as artificial analogues of proteins. Developing processes that grant access to such precision (bio)polymers will open the door to a vast unexplored area from which functional systems will be identified. A strong basis toward this endeavour was made using multicomponent peptide assemblies.^215,216^

**Fig. 17:**
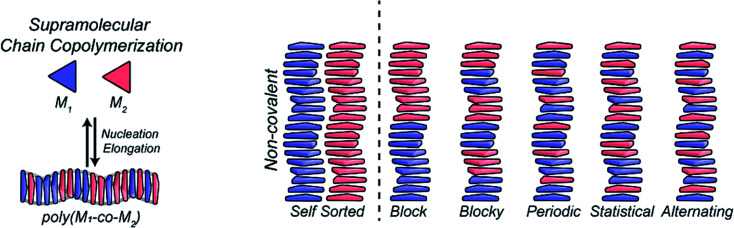
General mechanism of formation and possible organization of two-component supramolecular co-polymers. Reproduced with permission from ref. 217 (copyright 2019 American Chemical Society).

Using statistically-distributed multi-component dynamic covalent polymers, we have recently shown that multi-component DCPs can display enhanced properties with improved cell internalization properties compared to their homomeric counterparts.^39^ Regarding multi-component supramolecular polymers,^217,218^ the group of Meijer pioneered the initial investigations on supramolecular copolymers using BTA-type cooperative supramolecular polymers, studying how the presence of different monomers affects the structure and mechanism of polymerization,^219^ and documenting supramolecular block copolymers that form under thermodynamic control.^220,221^ The molecular structure of the aromatic core and of the peripheral groups is central here as it affects the propensity to polymerize and therefore the competition between self-polymerization *versus* co-polymerization (Fig. 17). For instance, peptides can be used as side-chains to enforce an alternating organization of supramolecular polymers dictated through electrostatic attractions.^156–158,222^

However, it is the recent discovery of living supramolecular polymerization that is really facilitating the preparation of supramolecular block copolymers, and several examples based on different molecular designs have been recently reported by the George,^223–226^ Würthner,^227^ and von Delius^58^ groups – all using two-component seeded living polymerization yielding very long fibres with a very limited length dispersity.

Finally, going towards precision DCPs at one building block resolution will require appropriate techniques to decipher and read their sequence.^228^ Compared to conventional “static” polymers, the unique feature of dynamic polymers (supramolecular polymers and DCPs) is that their sequence is not necessarily only programmed during synthesis but it can also be the result of a spontaneous self-selection enforced by folding, or of an adaptive sequence-selection due to binding to a templating (bio)molecular target.^229^ Looking in this direction, we believe the recent discovery reported by the groups of Huc and Otto of a self-folded low-symmetry monodisperse DCP^206^ represents a milestone that will prompt further exploration of multi-component DCPs. However, further development of our understanding of complex self-assembly processes leading to multi-component supramolecular polymers is necessary since a designed comonomer can practically turn into a chain capper, a sequestrator, an intercalator, or a comonomer – understanding how to navigate this fine line is therefore important.^230–232^

### 5.2. Transient supramolecular polymers

Many biological supramolecular polymers, such as actin filaments and microtubules,^149^ are temporally expressed, just at the place and for the time their function is needed. Thus, controlling the transient formation of supramolecular polymers has become a current goal of interest in the quest for man-made biomimetic systems.^233^

Supramolecular polymers may feature a diversity of molecular, supramolecular, and nano-scale organizations. However, since the systems are dynamic, interconversion is possible and all the different types of supramolecular polymers may influence each other like in a dynamic constitutional network.^146,234–236^ Interestingly, the so-called “off-pathway” supramolecular polymers formed under kinetic control may also display distinct and temporally-controlled properties such as chiral helical structures^237^ or fluorescence output.^61^

**Fig. 18:**
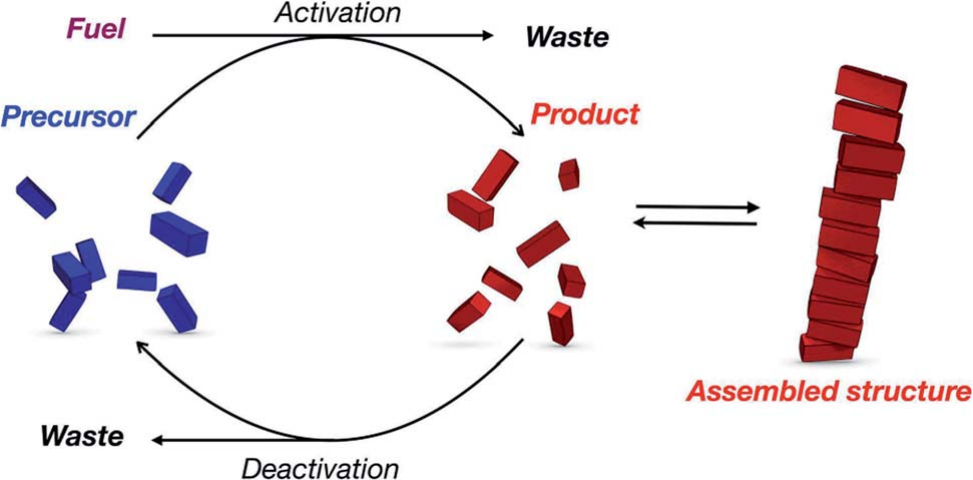
General mechanism representing the transient formation of supramolecular polymers by dissipative self-assembly (reproduced from ref. 243 with permission from John Wiley and Sons).

On the other hand, dissipative self-assemblies that consume a (bio)chemical fuel to sustain the formation of supramolecular polymers which subsequently collapse past its complete consumption also represent a powerful route toward transient materials (Fig. 18).^238,239^ In this regard, self-assemblies made of peptides are of particular interest since enzymatic^240^ or non-enzymatic processes (*e.g.* ester formation/hydrolysis, imine formation, and thiol–disulphide exchange/thiol–ester exchange)^241^ can perform transient modifications in aqueous media.^242–244^ For instance, using aromatic peptide amphiphiles, the group of Ulijn has precisely shown, in pioneering contributions,^245–248^ that protease-catalysed reactions can be used to form self-assembled nanostructures under biocatalytic control. This approach facilitates moving out-of-equilibrium and controlling the self-assembly pathways and emergence of material properties in space and time.^249,250^ The group also studied NDI and 1,8-naphthalimide (NI) amino acid methyl esters that are biocatalytically coupled to amino acid-amides using α-chymotrypsin^251,252^ and found nanotubes and nanofibers whose life-time is finely dictated by the nature of the amino acids ranging from minutes to hours, with a proof-of-concept showing the transient self-assembly of electronic wires in aqueous media. The George group reported several examples of dissipative supramolecular polymers using chemical fuels. For instance, a bisfunctionalized NDI compound was shown to undergo transient helical supramolecular polymerization fuelled by ATP, while the simultaneous presence of alkaline phosphatase (ALP) triggered its depolymerization.^253–255^ Redox control using Na_2_S_2_O_4_/O_2_ (ref. 256) over the folding of a NDI-pyrene donor–acceptor complex was also exploited for making supramolecular polymers with low dispersity.^257^

Here also, the combination of supramolecular and dynamic covalent chemistries is a fruitful approach leading to the discovery of new systems and new functions. The George group recently reported dissipative systems derived from their dormant charge-transfer monomer which turns into an activated amphiphile upon imine formation.^258^ The growth kinetics of supramolecular polymerization was found to be affected by the presence of inhibitors acting as a direct competitor of supramolecular polymerization, such as ethanol amine or urea/urease which produces ammonia *in situ*. On the other hand, lipase was found to promote depolymerization. Thus, this work shows that fine control of dynamic covalent self-assemblies may lead to supramolecular polymers with temporal control. Besides imine reactions, disulphides can also be used in dissipative systems. For instance, the Otto group used NaBO_3_ and tris(2-carboxyethyl)phosphine (TCEP) to, respectively, oxidize thiols into disulphides and reduce disulphides back into thiols, thus enabling self-replicating systems to operate out-of-equilibrium.^259^

Achieving precise control over the kinetics of assembly and disassembly that can be set by molecular engineering for a specific application is an important future line of development. In the long term, it remains an important challenge to correlate such dissipative self-assembly with a function.

## 6. Conclusions

This review discussed the recent merging of two areas that previously grew from different research communities: peptide-based materials and supramolecular polymers. In this study, we highlighted how modified amino acids and peptides can be inserted into dynamic covalent polymers for bringing functional groups and structured elements within these biomaterials. There is no doubt that new applications will be explored using bioconjugated dynamic covalent polymers. Also, an emerging, yet still challenging, direction is the self-selection of sequence-specific monodisperse dynamic covalent polymers from complex mixtures. In this review, we then described examples showing how β-sheet formation can be assisted by π–π stacking interactions for generating biomaterials such as hydrogels from simple aromatic-peptide conjugates. Conversely, we discussed recent achievements showing how peptide side-groups can influence the supramolecular polymerization of aromatics, enabling water-solubility, affecting their polymerization mechanism, bringing chiral information that can be transmitted to the supramolecular organization, and introducing, in a straightforward manner, reactive groups for developing functional supramolecular polymers. Finally, we highlighted recent advances in combining dynamic covalent chemistry with supramolecular polymerization of aromatic-peptide conjugates for obtaining biosupramolecular polymers in a hierarchical manner through complex self-assembly pathways through, for instance self-replication and triggered living polymerization.^260^ Current endeavours focus on multi-component precision polymers and on the transient expression of supramolecular polymers.

## 7. Perspectives

Peptide-based systems experience continuous and impressive developments as bioinspired soft materials for a wide range of applications (*e.g.* smart multivalent recognition and delivery, biomimetic catalysis, optics, bioelectronics, and biosensing),^261^ and a bright future lies ahead. The combination of peptide self-assembly with the self-assembly of aromatics brings a whole new flavour to the field, where aromatics can strengthen the peptide-based self-assembly and also bring new functions, whereas peptides can conversely contribute to the self-organization in aqueous media of the aromatic self-assemblies into chiral responsive nano-structures. Furthermore, new methodologies have been recently introduced that further boost the potential of the field: dynamic covalent chemistry and (living) supramolecular polymerization. While the former enables the covalent yet dynamic coupling of aromatics and peptides through covalent bonds that may be addressable, the latter grants access to multi-component biosupramolecular polymers by either integrative co-assembly of different building blocks,^218^ kinetic self-sorting in block copolymers,^227,262^ or sequential seeded-growth.^223^ Also, some of these systems can now form transiently under dissipative conditions. These trends underscore the pursuit toward life-like materials that are self-organized from simple building blocks into well-defined functional architectures through a spontaneous, dynamic, and adaptive hierarchical process, similarly to many examples of biopolymers such as microtubules which are part of the cytoskeleton of cells.^149^ Alternatively, there is no doubt that our ability to bring, in a defined order, multiple functions within a single yet multi-responsive chemical entity will unleash new properties, and hopefully technologies, possibly as bioactive molecules, catalysts, and sensors. For instance, the fine control over the complex dynamic self-assembly pathways described herein will subsequently enable control of, in space and in time, possibly also as a function of an internal or external chemical template,^263^ chemical fuel^52,239,264^ or a physical effector such as light,^265^ the dynamic expression of multivalent interactions that is crucial in the well-defined and controllable emergence of many functions (*e.g.* bioactivity and catalysis). As an illustrative example, one may cite here the contribution of the group of Pal which recently described aromatic peptide–bioglass nanocomposites, with a templated pathway-encoded fine structure, for application as bone scaffolds in tissue engineering.^266^ Thus, in conjunction with the ability of peptides to generate self-assemblies in complex biological environments,^267^ it appears clear that peptide-based supramolecular polymers will be central in the future development of supramolecular chemical biology.^268,269^ However, the achievements described in this review represent the first step toward this grand objective and many challenges are yet to be addressed, for instance, the stability and biocompatibility of complex self-assemblies at low concentrations in biological fluids.

## Author contributions

All authors discussed the scope, the organization of the manuscript and the selection of its content. All authors contributed to writing the initial draft, drawing the illustrations, reviewing and editing.

## Conflicts of interest

There are no conflicts to declare.

## Supplementary Material
